# *N*-(4-Bromo­benzyl­idene)-3,4-dimethyl­isoxazol-5-amine

**DOI:** 10.1107/S1600536810027893

**Published:** 2010-07-21

**Authors:** Abdullah M. Asiri, Salman A. Khan, M. Nawaz Tahir

**Affiliations:** a The Center of Excellence for Advanced Materials Research, King Abdul Aziz University, Jeddah 21589, PO Box 80203, Saudi Arabia; b Department of Chemistry, Faculty of Science, King Abdul Aziz University, Jeddah 21589, PO Box 80203, Saudi Arabia; c Department of Physics, University of Sargodha, Sargodha, Pakistan

## Abstract

In the title compound, C_12_H_11_BrN_2_O, the 4-bromo­benzaldehyde and 5-amino-3,4-dimethyl­isoxazole units are oriented at a dihedral angle of 4.89 (8)°. In the crystal, weak π–π inter­actions are present between the benzene rings at a centroid–centroid distance of 3.7862 (14) Å.

## Related literature

For related structures, see: Asiri *et al.* (2010 ▶): Fun *et al.* (2010*a* ▶,*b* ▶): Shad *et al.* (2008 ▶): Tahir *et al.* (2008 ▶). For graph-set notation, see: Bernstein *et al.* (1995 ▶).
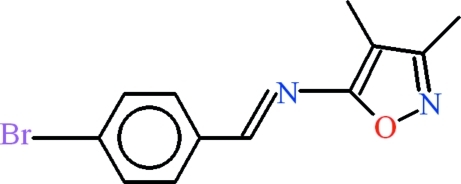


## Experimental

### 

#### Crystal data

C_12_H_11_BrN_2_O
*M* *_r_* = 279.14
Triclinic, 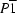

*a* = 7.6406 (4) Å
*b* = 8.8709 (5) Å
*c* = 9.1052 (5) Å
α = 97.024 (2)°
β = 102.961 (1)°
γ = 92.786 (2)°
*V* = 595.06 (6) Å^3^
*Z* = 2
Mo *K*α radiation
μ = 3.43 mm^−1^
*T* = 296 K
0.30 × 0.14 × 0.12 mm

#### Data collection

Bruker Kappa APEXII CCD diffractometer
Absorption correction: multi-scan (*SADABS*; Bruker, 2005 ▶) *T*_min_ = 0.568, *T*_max_ = 0.665
8212 measured reflections
2119 independent reflections
1643 reflections with *I* > 2σ(*I*)
*R*_int_ = 0.022

#### Refinement

*R*[*F* ^2^ > 2σ(*F* ^2^)] = 0.025
*wR*(*F* ^2^) = 0.059
*S* = 1.03
2119 reflections
147 parameters
H-atom parameters constrained
Δρ_max_ = 0.20 e Å^−3^
Δρ_min_ = −0.17 e Å^−3^

### 

Data collection: *APEX2* (Bruker, 2009 ▶); cell refinement: *SAINT* (Bruker, 2009 ▶); data reduction: *SAINT*; program(s) used to solve structure: *SHELXS97* (Sheldrick, 2008 ▶); program(s) used to refine structure: *SHELXL97* (Sheldrick, 2008 ▶); molecular graphics: *ORTEP-3 for Windows* (Farrugia, 1997 ▶) and *PLATON* (Spek, 2009 ▶); software used to prepare material for publication: *WinGX* (Farrugia, 1999 ▶) and *PLATON*.

## Supplementary Material

Crystal structure: contains datablocks global, I. DOI: 10.1107/S1600536810027893/bq2226sup1.cif

Structure factors: contains datablocks I. DOI: 10.1107/S1600536810027893/bq2226Isup2.hkl

Additional supplementary materials:  crystallographic information; 3D view; checkCIF report

## Figures and Tables

**Fig. 1.**
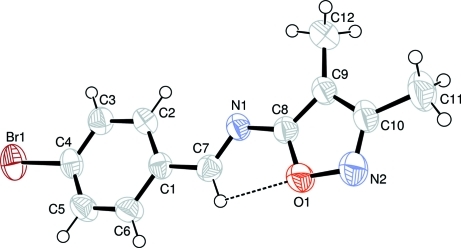
View of the title compound with the atom numbering scheme. The thermal ellipsoids are drawn at the 50% probability level. The dotted line indicate the intramolecular H-bond.

## supplementary crystallographic information

### Comment

Heterocycles such as nitrogen and oxygen containing compounds are abundant in nature and are of great significance to life. We herein report the synthesis and crystal structure of title compound (I, Fig. 1).

The crystal structures of 4-chloro-2- [(*E*)-({4-[*N*-(3,4-dimethylisoxazol-5-yl)sulfamoyl]phenyl}iminio) methyl]phenolate (Shad *et al.*, 2008), 4-bromo-2-((*E*)-{4-[(3,4-dimethylisoxazol-5-yl)sulfamoyl]phenyl} iminiomethyl)phenolate (Tahir *et al.*, 2008), 2-[(*E*)-(3,4-dimethylisoxazol-5-yl)iminomethyl]phenol (Fun *et al.*, 2010*a*), 1-[(*E*)-(3,4-dimethylisoxazol-5-yl)iminomethyl]-2-naphthol (Fun *et al.*, 2010*b*) and *N*-[4-(dimethylamino)benzylidene]-3,4-dimethylisoxazol-5-amine (Asiri *et al.*, 2010) have been published previously, which contain the 5-amino-3,4-dimethylisoxazole moiety.

In (I), the 4-bromobenzaldehyde moiety A (C1—C7/BR1) and 5-amino-3,4-dimethylisoxazole moiety B (N1/C8—C12/N2/O1) are planar with r. m. s. deviations of 0.0119 Å and 0.0128 Å, respectively. The dihedral angle between A/B is 4.89 (8)°. The title compound essentially consists of monomers. Weak intramolecular H-bonding of C—H···O type (Table 1, Fig. 1) exists and complete an S(5) ring motif (Bernstein *et al.*, 1995). There exists also π–π interaction between the centroids of phenyl rings at a distance of 3.7862 (14) Å [symmetry code: -*x*, 2 - *y*, 1 - *z*].

### Experimental

A mixture of 4-bromobenzaldehyde (0.40 g, 0.0022 mol) and 5-amino-3,4-dimethylisoxazole (0.24 g, 0.0022 mol) in ethanol (15 ml) was refluxed for 5 h with stirring to give a light brown needles of title compound (I).

### Refinement

The H-atoms were positioned geometrically (C–H = 0.93–0.96 Å) and refined as riding with *U*_iso_(H) = x*U*_eq_(C), where x = 1.5 for methyl and x = 1.2 for aryl H-atoms.

### Crystal data

**Table:**

C_12_H_11_BrN_2_O	*Z* = 2
*M**_r_* = 279.14	*F*(000) = 280
Triclinic, *P*1	*D*_x_ = 1.558 Mg m^−^^3^
Hall symbol: -P 1	Mo *K*α radiation, λ = 0.71073 Å
*a* = 7.6406 (4) Å	Cell parameters from 1643 reflections
*b* = 8.8709 (5) Å	θ = 2.3–25.3°
*c* = 9.1052 (5) Å	µ = 3.43 mm^−^^1^
α = 97.024 (2)°	*T* = 296 K
β = 102.961 (1)°	Needle, brown
γ = 92.786 (2)°	0.30 × 0.14 × 0.12 mm
*V* = 595.06 (6) Å^3^	

### Data collection

**Table:**

Bruker Kappa APEXII CCD diffractometer	2119 independent reflections
Radiation source: fine-focus sealed tube	1643 reflections with *I* > 2σ(*I*)
graphite	*R*_int_ = 0.022
Detector resolution: 8.10 pixels mm^-1^	θ_max_ = 25.3°, θ_min_ = 2.3°
ω scans	*h* = −9→9
Absorption correction: multi-scan (*SADABS*; Bruker, 2005)	*k* = −10→10
*T*_min_ = 0.568, *T*_max_ = 0.665	*l* = −10→10
8212 measured reflections	

### Refinement

**Table:**

Refinement on *F*^2^	Primary atom site location: structure-invariant direct methods
Least-squares matrix: full	Secondary atom site location: difference Fourier map
*R*[*F*^2^ > 2σ(*F*^2^)] = 0.025	Hydrogen site location: inferred from neighbouring sites
*wR*(*F*^2^) = 0.059	H-atom parameters constrained
*S* = 1.03	*w* = 1/[σ^2^(*F*_o_^2^) + (0.0225*P*)^2^ + 0.2246*P*] where *P* = (*F*_o_^2^ + 2*F*_c_^2^)/3
2119 reflections	(Δ/σ)_max_ = 0.001
147 parameters	Δρ_max_ = 0.20 e Å^−^^3^
0 restraints	Δρ_min_ = −0.17 e Å^−^^3^

### Special details

**Table:**

Geometry. Bond distances, angles *etc*. have been calculated using the rounded fractional coordinates. All su's are estimated from the variances of the (full) variance-covariance matrix. The cell e.s.d.'s are taken into account in the estimation of distances, angles and torsion angles
Refinement. Refinement of *F*^2^ against ALL reflections. The weighted *R*-factor *wR* and goodness of fit *S* are based on *F*^2^, conventional *R*-factors *R* are based on *F*, with *F* set to zero for negative *F*^2^. The threshold expression of *F*^2^ > σ(*F*^2^) is used only for calculating *R*-factors(gt) *etc*. and is not relevant to the choice of reflections for refinement. *R*-factors based on *F*^2^ are statistically about twice as large as those based on *F*, and *R*- factors based on ALL data will be even larger.

### Fractional atomic coordinates and isotropic or equivalent isotropic displacement parameters (Å^2^)

**Table:**

	*x*	*y*	*z*	*U*_iso_*/*U*_eq_	
Br1	−0.32255 (4)	1.00984 (3)	0.72075 (3)	0.0712 (1)	
O1	0.4539 (2)	0.51578 (18)	0.30328 (18)	0.0572 (6)	
N1	0.1487 (3)	0.55019 (19)	0.3071 (2)	0.0440 (6)	
N2	0.5503 (3)	0.4289 (3)	0.2111 (3)	0.0656 (8)	
C1	0.0706 (3)	0.7262 (2)	0.4987 (2)	0.0421 (8)	
C2	−0.1140 (3)	0.7008 (2)	0.4413 (3)	0.0471 (8)	
C3	−0.2317 (3)	0.7844 (3)	0.5070 (3)	0.0509 (8)	
C4	−0.1622 (3)	0.8940 (2)	0.6301 (3)	0.0489 (9)	
C5	0.0186 (4)	0.9215 (3)	0.6888 (3)	0.0571 (9)	
C6	0.1356 (3)	0.8365 (3)	0.6240 (3)	0.0538 (9)	
C7	0.1988 (3)	0.6429 (2)	0.4298 (3)	0.0455 (8)	
C8	0.2749 (3)	0.4794 (2)	0.2430 (3)	0.0433 (8)	
C9	0.2501 (3)	0.3744 (2)	0.1180 (3)	0.0457 (8)	
C10	0.4265 (3)	0.3478 (3)	0.1044 (3)	0.0517 (9)	
C11	0.4836 (4)	0.2423 (3)	−0.0149 (3)	0.0745 (11)	
C12	0.0764 (4)	0.3028 (3)	0.0200 (3)	0.0659 (10)	
H2	−0.15915	0.62688	0.35786	0.0565*	
H3	−0.35551	0.76687	0.46887	0.0611*	
H5	0.06270	0.99657	0.77139	0.0686*	
H6	0.25905	0.85335	0.66457	0.0646*	
H7	0.32075	0.65805	0.47691	0.0546*	
H11A	0.61269	0.24881	0.00435	0.1118*	
H11B	0.43483	0.27080	−0.11341	0.1118*	
H11C	0.43975	0.13964	−0.01207	0.1118*	
H12A	−0.02153	0.34332	0.05809	0.0989*	
H12B	0.07209	0.19462	0.02086	0.0989*	
H12C	0.06658	0.32433	−0.08221	0.0989*	

### Atomic displacement parameters (Å^2^)

**Table:**

	*U*^11^	*U*^22^	*U*^33^	*U*^12^	*U*^13^	*U*^23^
Br1	0.0760 (2)	0.0641 (2)	0.0851 (2)	0.0188 (1)	0.0448 (2)	0.0012 (1)
O1	0.0487 (11)	0.0650 (10)	0.0542 (10)	0.0090 (8)	0.0134 (8)	−0.0109 (8)
N1	0.0499 (12)	0.0408 (10)	0.0421 (11)	0.0068 (8)	0.0138 (9)	0.0023 (9)
N2	0.0549 (14)	0.0742 (14)	0.0684 (15)	0.0155 (11)	0.0226 (12)	−0.0074 (12)
C1	0.0501 (15)	0.0399 (12)	0.0394 (13)	0.0059 (10)	0.0163 (10)	0.0061 (10)
C2	0.0523 (16)	0.0422 (12)	0.0466 (14)	0.0001 (10)	0.0157 (11)	−0.0006 (10)
C3	0.0448 (15)	0.0493 (13)	0.0603 (16)	0.0033 (10)	0.0164 (12)	0.0063 (12)
C4	0.0598 (17)	0.0419 (12)	0.0521 (15)	0.0077 (10)	0.0271 (12)	0.0063 (11)
C5	0.0624 (19)	0.0568 (15)	0.0492 (15)	0.0015 (12)	0.0174 (12)	−0.0115 (12)
C6	0.0456 (15)	0.0632 (15)	0.0486 (15)	0.0020 (11)	0.0104 (11)	−0.0063 (12)
C7	0.0449 (14)	0.0482 (13)	0.0446 (14)	0.0075 (10)	0.0117 (10)	0.0071 (11)
C8	0.0476 (15)	0.0428 (12)	0.0410 (13)	0.0081 (10)	0.0119 (10)	0.0065 (10)
C9	0.0590 (16)	0.0397 (12)	0.0398 (13)	0.0087 (10)	0.0140 (11)	0.0045 (10)
C10	0.0652 (17)	0.0456 (13)	0.0482 (15)	0.0143 (12)	0.0209 (13)	0.0037 (11)
C11	0.089 (2)	0.0725 (18)	0.0698 (19)	0.0249 (15)	0.0379 (16)	−0.0036 (14)
C12	0.073 (2)	0.0616 (16)	0.0553 (16)	0.0060 (13)	0.0070 (14)	−0.0075 (13)

### Geometric parameters (Å, °)

**Table:**

Br1—C4	1.899 (2)	C9—C10	1.409 (3)
O1—N2	1.420 (3)	C9—C12	1.486 (4)
O1—C8	1.361 (3)	C10—C11	1.500 (4)
N1—C7	1.274 (3)	C2—H2	0.9300
N1—C8	1.374 (3)	C3—H3	0.9300
N2—C10	1.307 (4)	C5—H5	0.9300
C1—C2	1.387 (3)	C6—H6	0.9300
C1—C6	1.390 (3)	C7—H7	0.9300
C1—C7	1.460 (3)	C11—H11A	0.9600
C2—C3	1.384 (3)	C11—H11B	0.9600
C3—C4	1.378 (4)	C11—H11C	0.9600
C4—C5	1.363 (4)	C12—H12A	0.9600
C5—C6	1.382 (4)	C12—H12B	0.9600
C8—C9	1.351 (3)	C12—H12C	0.9600
			
Br1···C11^i^	3.595 (3)	C4···C6^ii^	3.553 (3)
Br1···C7^ii^	3.687 (2)	C6···C4^ii^	3.553 (3)
O1···C3^iii^	3.355 (3)	C7···Br1^ii^	3.687 (2)
O1···H3^iii^	2.6900	C7···C2^iv^	3.481 (3)
O1···H7	2.3400	C8···C3^iv^	3.512 (3)
N1···C2^iv^	3.426 (3)	C11···Br1^viii^	3.595 (3)
N1···H2	2.6000	H2···N1	2.6000
N1···H12A	2.7600	H2···N2^vii^	2.7400
N1···H12C^v^	2.7100	H3···O1^vii^	2.6900
N2···H2^iii^	2.7400	H6···H7	2.4200
N2···H11B^vi^	2.9100	H7···O1	2.3400
C2···N1^iv^	3.426 (3)	H7···H6	2.4200
C2···C7^iv^	3.481 (3)	H11B···N2^vi^	2.9100
C3···O1^vii^	3.355 (3)	H12A···N1	2.7600
C3···C8^iv^	3.512 (3)	H12C···N1^v^	2.7100
			
N2—O1—C8	107.76 (18)	C1—C2—H2	120.00
C7—N1—C8	119.9 (2)	C3—C2—H2	120.00
O1—N2—C10	105.0 (2)	C2—C3—H3	121.00
C2—C1—C6	118.9 (2)	C4—C3—H3	121.00
C2—C1—C7	122.08 (18)	C4—C5—H5	120.00
C6—C1—C7	119.0 (2)	C6—C5—H5	120.00
C1—C2—C3	120.6 (2)	C1—C6—H6	120.00
C2—C3—C4	118.8 (2)	C5—C6—H6	120.00
Br1—C4—C3	119.17 (18)	N1—C7—H7	119.00
Br1—C4—C5	119.02 (19)	C1—C7—H7	119.00
C3—C4—C5	121.8 (2)	C10—C11—H11A	109.00
C4—C5—C6	119.2 (2)	C10—C11—H11B	109.00
C1—C6—C5	120.6 (2)	C10—C11—H11C	109.00
N1—C7—C1	122.0 (2)	H11A—C11—H11B	109.00
O1—C8—N1	120.5 (2)	H11A—C11—H11C	109.00
O1—C8—C9	110.4 (2)	H11B—C11—H11C	109.00
N1—C8—C9	129.2 (2)	C9—C12—H12A	109.00
C8—C9—C10	103.9 (2)	C9—C12—H12B	109.00
C8—C9—C12	127.6 (2)	C9—C12—H12C	109.00
C10—C9—C12	128.5 (2)	H12A—C12—H12B	110.00
N2—C10—C9	113.0 (2)	H12A—C12—H12C	109.00
N2—C10—C11	118.9 (2)	H12B—C12—H12C	109.00
C9—C10—C11	128.1 (2)		
			
C8—O1—N2—C10	−0.1 (3)	C1—C2—C3—C4	0.3 (4)
N2—O1—C8—C9	0.1 (2)	C2—C3—C4—Br1	179.81 (18)
N2—O1—C8—N1	−178.1 (2)	C2—C3—C4—C5	−0.3 (4)
C7—N1—C8—C9	177.3 (2)	Br1—C4—C5—C6	179.42 (19)
C8—N1—C7—C1	177.18 (18)	C3—C4—C5—C6	−0.5 (4)
C7—N1—C8—O1	−4.8 (3)	C4—C5—C6—C1	1.2 (4)
O1—N2—C10—C9	0.1 (3)	O1—C8—C9—C10	0.0 (3)
O1—N2—C10—C11	179.6 (2)	N1—C8—C9—C12	−2.8 (4)
C2—C1—C6—C5	−1.2 (3)	O1—C8—C9—C12	179.1 (2)
C6—C1—C2—C3	0.4 (3)	N1—C8—C9—C10	178.0 (2)
C7—C1—C2—C3	−178.4 (2)	C8—C9—C10—N2	−0.1 (3)
C7—C1—C6—C5	177.6 (2)	C12—C9—C10—C11	1.4 (4)
C2—C1—C7—N1	5.0 (3)	C8—C9—C10—C11	−179.5 (3)
C6—C1—C7—N1	−173.7 (2)	C12—C9—C10—N2	−179.2 (2)

Symmetry codes: (i) *x*−1, *y*+1, *z*+1; (ii) −*x*, −*y*+2, −*z*+1; (iii) *x*+1, *y*, *z*; (iv) −*x*, −*y*+1, −*z*+1; (v) −*x*, −*y*+1, −*z*; (vi) −*x*+1, −*y*+1, −*z*; (vii) *x*−1, *y*, *z*; (viii) *x*+1, *y*−1, *z*−1.

### Hydrogen-bond geometry (Å, °)

**Table:**

*D*—H···*A*	*D*—H	H···*A*	*D*···*A*	*D*—H···*A*
C7—H7···O1	0.9300	2.3400	2.702 (3)	103.00
